# Micromechanical Characterization of Polysilicon Films through On-Chip Tests

**DOI:** 10.3390/s16081191

**Published:** 2016-07-28

**Authors:** Ramin Mirzazadeh, Saeed Eftekhar Azam, Stefano Mariani

**Affiliations:** Dipartimento di Ingegneria Civile e Ambientale, Politecnico di Milano, Piazza L. da Vinci 32, 20133 Milano, Italy; ramin.mirzazadeh@polimi.it (R.M.); saeed.eftekhar@polimi.it (S.E.A.)

**Keywords:** MEMS, polysilicon film, on-chip test, over-etch variation, particle filtering, reduced-order modeling

## Abstract

When the dimensions of polycrystalline structures become comparable to the average grain size, some reliability issues can be reported for the moving parts of inertial microelectromechanical systems (MEMS). Not only the overall behavior of the device turns out to be affected by a large scattering, but also the sensitivity to imperfections gets enhanced. In this work, through on-chip tests, we experimentally investigate the behavior of thin polysilicon samples using standard electrostatic actuation/sensing. The discrepancy between the target and actual responses of each sample has then been exploited to identify: (i) the overall stiffness of the film and, according to standard continuum elasticity, a morphology-based value of its Young’s modulus; (ii) the relevant over-etch induced by the fabrication process. To properly account for the aforementioned stochastic features at the micro-scale, the identification procedure has been based on particle filtering. A simple analytical reduced-order model of the moving structure has been also developed to account for the nonlinearities in the electrical field, up to pull-in. Results are reported for a set of ten film samples of constant slenderness, and the effects of different actuation mechanisms on the identified micromechanical features are thoroughly discussed.

## 1. Introduction

Even though microelectromechanical systems (MEMS) have proven to be potential candidates for a wide range of applications, such as gyroscopes, accelerometers, biosensors, switches and magnetometers, the design uncertainties are still a challenging issue when new devices have to be designed. These uncertainties are recognized to be of crucial importance to the performance of MEMS; a family of in situ methods has been therefore developed in the MEMS design community to adjust the mechanical stiffness of deformable components and, so, compensate for the performance inaccuracies after fabrication; see, e.g., [1]. The advances in the microfabrication process and the continuous demand of miniaturization may amplify the impact of these uncertainties when the reliability and reproducibility of the systems are of concern.

The sources of the aforementioned uncertainties can be either intrinsic, like material properties, or extrinsic, like process-induced variations in the geometry. Since the structural components of moving parts can have a characteristic size comparable to a kind of material length-scale, linked, e.g., to the grain size in polysilicon films, the relevant mechanical properties can not be defined deterministically. While for single-crystalline silicon, the elastic properties have been accurately correlated to the relative orientation of the crystal lattice and the testing direction, for polysilicon materials, the grain morphology has been shown to have a strong impact on the same properties. In [2], the Young’s modulus of specimens made of columnar polysilicon with a grain size of 0.2–0.5 μm was shown to have a mean value of around 158 GPa, with a significant scattering (featuring a standard deviation of about 10 GPa) as the size of the specimen was decreased down to 6 μm. The same Young’s modulus was reported in [3] to be 147 GPa, with a scattering of 10%–15% for specimens of a length ranging between 5 and 200 μm and a width between 1 and 6 μm. This scattering can be either due to the errors in the measurements or to the fact that the number of grains in the specimen is not enough to consider the polysilicon film statistically representative [4,5]. There have been other studies showing minor discrepancies, on the order of a few GPa, for slender specimens tested in tension [6,7,8]; however, it should be noticed that such results can be achieved only in the absence of significant stress and strain gradients in the tested samples. Considering the wide use of bending modes for sensing applications, which all lead to a non-uniform stress/strain profile, results are supposed to be more sensitive to surface effects [9].

An additional source of uncertainties at the film level is the variation in the over-etch [10], i.e., the surplus of etch with respect to the expected one. According to [11], “an etch variation defect is one that the thickness of the device structure does not meet the design expectation due to the etch variations caused by fluctuation of temperature, etchant concentration, and other reasons”. Though the order of such variation is less than 1 μm (depending on the production process and on the geometry), its relative importance can increase when the components shrink down to a comparable size. Overall, a variation of 10% in the geometry can be expected [12]. In [13], capacitive gaps of 5.3 and 5.6 μm were reported for a layout value of 5 μm; in [14], it was stated that for a 2 μm-thick structural polysilicon, patterned by a wafer stepper and etched with a reactive-ion etcher, a reasonable estimate for the variation in the linear dimension of etched features was about 0.2 μm (with 10% relative tolerance). These variations have dominant effects on the bending rigidity of beams, due to variations of the in-plane thickness, or on the sensing and actuation mechanisms, due to variations of the gap at capacitors.

To characterize the effects of these two sources of uncertainties on the electro-mechanical response of MEMS devices, several methods have been proposed in the literature. A class of deterministic methods is based on the surface inspection of the devices through SEM imaging or laser interferometry [15,16,17]; these methods are not only slow to perform, but also limited to devices on open wafers, while commercial ones are usually packed to protect the system from the environment. Other methods use embedded structures for device characterization [11,18,19]. The deviation of the pull-in voltage from the expected value can be also adopted for process characterization [20,21,22]; however, the pull-in value is very sensitive to the accuracy of the model. Alternative stochastic methods are instead based on, e.g., Monte Carlo and spectral simulations and stochastic collocation; see [23,24,25,26,27].

In this work, a simple on-chip test is proposed to assess the variations of the over-etch and overall stiffness property of the deforming structure. The movable part of the device is made of polysilicon, obtained through epitaxial growth with a direction almost perpendicular to the substrate and with an average grain size of 500 nm. The specimen subject to deformation is a 20 μm-long microcantilever, featuring a 2 μm × 22 μm rectangular cross-section. Sensing and actuation are obtained using a standard electrostatic method: thanks to the combined use of different stators deployed around the movable proof mass, two different sensing modes are exploited to characterize the mechanical response of the film under two different bending-dominated loading conditions. The tested devices have shown a significant scattering in the experimentally-measured electro-mechanical response, even at constant nominal properties. To infer the film properties, an analytical model for the electro-mechanical behavior of the system has been developed. Next, particle filtering has been adopted to get the values of over-etch and Young’s modulus for each tested device, based on the measured capacitance change vs. applied voltage. Instead of using the pull-in value alone, which is here shown not to vary monotonically with the over-etch for the considered geometry, a Bayesian update is resorted to get insights from the whole response of the device at increasing voltage up to (or close to) pull-in.

The remainder of this article is organized as follows. In Section 2, we present the details of the designed test and the experimental results. In Section 3, the analytical reduced-order model of the test structure is detailed. The adopted particle filter used for parameter identification purposes is explained in Section 4. Major results of the proposed method are discussed in Section 5. Finally, some concluding remarks and proposals for future works are collected in Section 6.

## 2. On-Chip Test Device: Operational Principles and Experimental Results

**Figure 1:**
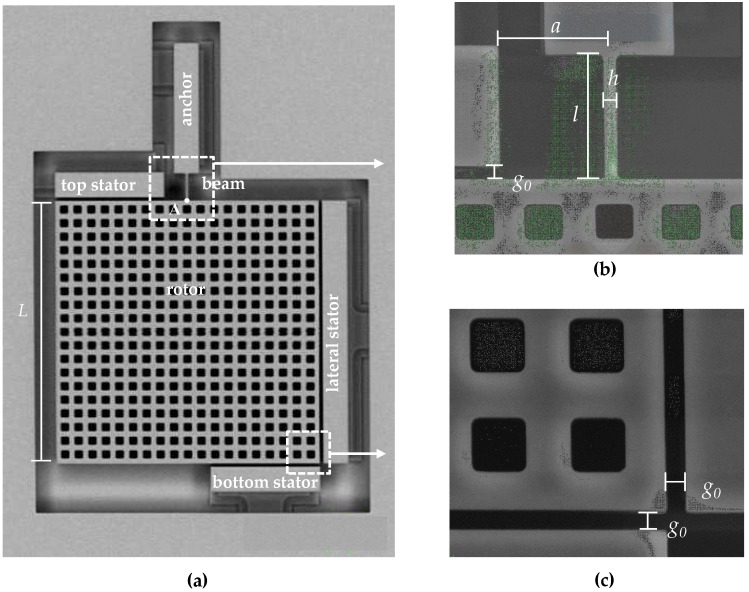
SEM picture of (**a**) the testing device; and close-ups of (**b**) the beam and of (**c**) the bottom-right corner of the plate.

The study has been carried out through the designed device reported in Figure 1, whose geometrical parameters are summarized in Table 1. The device has been fabricated with the THELMA*©* (Thick Epipoly Layer for Microactuators and Accelerometers) surface micromachining process developed by STMicroelectronics [28], with an out-of-plane thickness *w* = 22 μm of the polysilicon film. Several devices have been patterned on a single wafer with identical nominal characteristics and packed at low pressure (100 Pa) with a wafer-to-wafer bonding technique. To ensure complete release of the moving structure from the substrate, holes have been incorporated in massive parts of the device (plate). The system is composed of three stators and a movable mass (denoted as rotor in the figure) connected to the anchor at the top side through a slender micro-beam. Disregarding possible compliant mechanisms at the anchor point, the beam behaves like a cantilever. Due to its geometry and anchoring condition, the structure results in being statically determinate; hence, the effects of residual stresses (if any) on the device response are supposed to be negligible. The anchor, the beam and the rotor are electrically grounded, while the top and bottom stators are electrically connected to one another. The alternate choice of the rotational stators or of the lateral one provides two different possibilities for both sensing and actuation and, so, overall, four different testing configurations.

In the case of lateral actuation, the rotor mainly undergoes a lateral displacement, but, due to the asymmetry induced by the beam, an additional rotation shows up. Overall, the beam is subjected to a mixed bending/shear deformation mode; due to its slenderness of l/h=10, shear effects have a marginal impact. In the case of rotational actuation induced simultaneously by the top and bottom stators, their symmetric positioning around the anchor point induces instead a pure rotation of the proof mass and, so, a pure bending deformation of the beam.

Sensing is obtained by measuring the capacitance change during the motion of the rotor; while the system actuation is realized with a bias voltage, an additional sinusoidal voltage with an amplitude of 200 mV and a frequency of 1 MHz is used for sensing at the capacitors.

**Figure 2:**
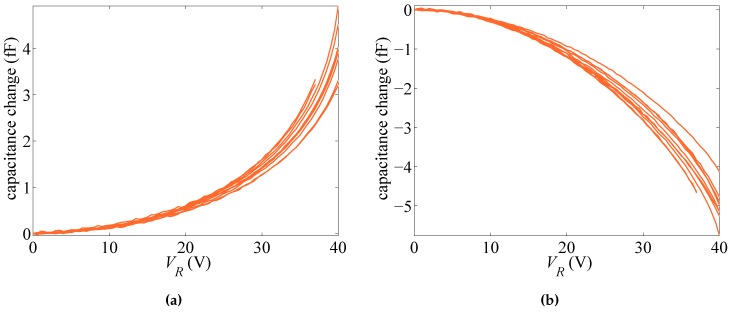
Rotational actuation through $V_{R}$: experimentally-measured capacitance change with (**a**) rotational sensing and (**b**) lateral sensing.

**Figure 3:**
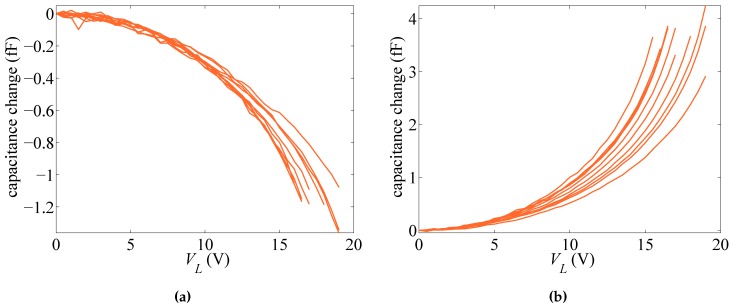
Lateral actuation through $V_{L}$: experimentally-measured capacitance change with (**a**) rotational sensing and (**b**) lateral sensing.

In what follows, we gather the results on the basis of the type of actuation. Accordingly, two different scenarios are obtained if rotational capacitors are biased by voltage $V_{R}$ or if the lateral capacitor is biased by voltage $V_{L}$. We therefore name the two cases as rotational and lateral actuation. The responses of ten different devices, in terms of the change in the capacitance values at increasing bias voltage for the rotational and lateral cases, are respectively reported in Figure 2 and Figure 3. Full loading and unloading measurements have been carried out for each device: since an excellent repeatability has been observed, with no hysteresis along loading and unloading cycles, only one measurement (loading) curve is reported for each device. Regarding the rotational case, the actuation has been increased up to 40 V only; since the target of the experimental tests is not the assessment of the pull-in instability, which can damage the functionality of the devices by the consequent electrical short-circuit, some devices that have more compliant responses have been actuated up to lower voltage levels. The same strategy has been also adopted for the lateral actuation. In the graphs, either positive or negative values of capacitance change are reported; the first ones appear when sensing and actuation are obtained with the same capacitor (i.e., for rotational sensing and actuation or lateral sensing and actuation), whereas the second ones when different capacitors are used. Positive values obviously mean that, due to the beam deformation, on average, a reduction of the gap with respect to the initial one has been induced; on the contrary, negative values mean that, once again on average, conductors are somehow moving away from one another. It can be also seen that the lateral sensing and actuation are more configurationally responsive, as pull-in is approached for values of the applied voltage $V_{L}$ amounting to around half of those necessary to approach instead pull-in under applied voltage $V_{R}$, measuring basically the same capacitance change. The effects of measurement noise are shown at low actuation levels (see, e.g., Figure 3a), but the measurement to error ratio improves as the level of actuation rises.

Figure 2 and Figure 3 show that the response of the devices is rather scattered. In [29], it was shown that such scattering cannot be linked to micromechanical features of the polysilicon film only; through a Monte Carlo analysis and finite element simulations of the coupled electro-mechanical response of the test structure, wherein the morphology of the polycrystalline silicon was appropriately handled, a by far less scattered response was foreseen. Hence, the different responses collected in the plots cannot be physically justified on purely mechanical grounds and must be induced by small differences in the configuration of the capacitors, as well. Accordingly, both the geometric features and the overall, or homogenized material Young’s modulus of the micro-beam (see [27]), are dealt with as stochastic variables in the forthcoming analysis.

## 3. Analytical Modeling of the Test Structure

As shown in Figure 1 and discussed in Section 2, the moving part of the structure is made of a plate, connected to the substrate through a slender beam that actually represents the tested specimen. Under static loading and even in the case of low-frequency dynamic excitations, the plate is assumed to undergo an in-plane, purely rigid motion triggered by either group of conductors.

During the experiments, only the input voltage-output capacitance change transfer function has been assessed for capped devices. In this section, we therefore develop a simplified, analytic electro-mechanical formulation able to catch the same function for the target reference geometry and also assess the spreading induced by variations of over-etch and the stiffness of the micro-beam.

**Figure 4:**
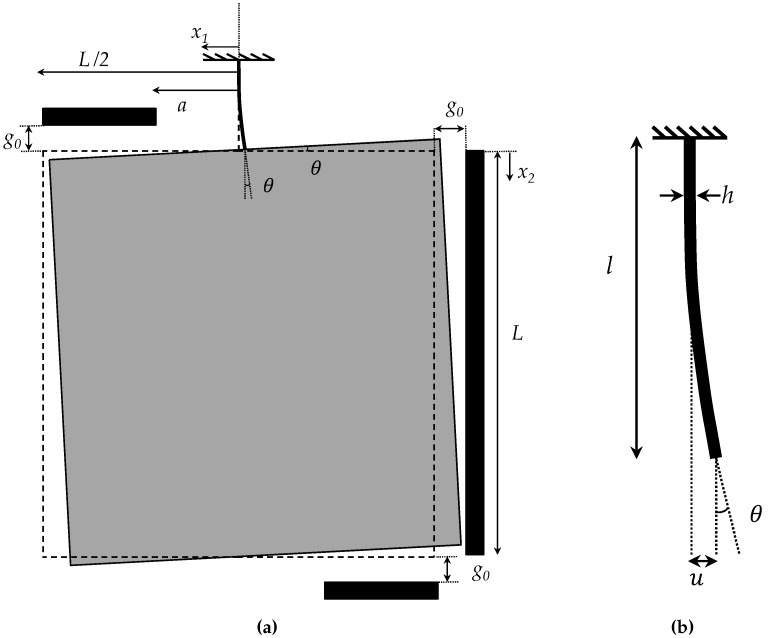
(**a**) Schematic of the behavior of the whole test structure in the case of lateral actuation and (**b**) close-up of the deflected micro-beam.

Due to its slenderness, the beam is modeled according to the Bernoulli-Euler theory, and shear deformations are neglected all over its length. When the proof mass undergoes any displacement, a load is transferred to the beam, with an in-plane moment *M* and a shear force *F* as components at the tip of the cantilever (Point A in Figure 1). Beam bending is governed by a fourth order ordinary differential equation, which, in the absence of axial and lateral distributed forces, can be solved analytically using polynomial functions. Given the constraints at the anchor side, the mechanical response of the beam can be formulated in terms of the following relationship between *F* and *M* and the lateral displacement *u* and rotation angle *θ* at the beam tip [30]; see Figure 4;

(1) $$\left\{\begin{array}{c} F\\ M \end{array}\right\}=\frac{EI}{l^{3}}\left[\begin{array}{cc} 12 & -6l\\ -6l & 4l^{2} \end{array}\right]\left\{\begin{array}{c} u\\ \theta \end{array}\right\}$$

where *l*, *h* and *w* are, respectively, the length, in-plane width and out-of plane thickness of the beam, $I=wh^{3}/12$ is the in-plane moment of inertia of the beam cross-section, *E* is the effective Young’s modulus of the polysilicon film and, thus, EI represents the flexural rigidity of the cantilever. In Equation (1), the behavior of the specimen has been considered linear, as the expected values of *u* and *θ* are not supposed to induce finite strains.

Values of *F* and *M* need now to be linked to the electrostatic actuation provided to the plate by the conductors. Moving from the case of a parallel plate capacitor, the attraction force per unit area between the conductors can be linked to the applied voltage *V* and the gap distance *g* according to [31]:

(2) $$f=\frac{\epsilon V^{2}}{2g^{2}}\;$$

where $\epsilon =\epsilon_{r}\epsilon_{o}$, $\epsilon_{r}$ is the relative permittivity of the medium and $\epsilon_{0}$ is the dielectric constant in the free space. Equation (2) clearly shows that *f* is inherently a nonlinear function of the gap *g* in the specific case of capacitor plates with surfaces parallel to each other. For the same configuration, the capacitance of the capacitor per unit area can be obtained as:

(3) $$c=\frac{\epsilon }{g}\;$$

By disregarding fringe field effects and assuming the rotation angle *θ* to be always small, the forcing terms *F* and *M* can be computed by integrating all over the stator surfaces the local contributions in terms of *f*. Since under actuation, the proof mass is displaced as a rigid body, the local gap *g* can be analytically linked to *u* and *θ*.

In what follows, we distinguish the rotational and lateral actuation cases, and we separately provide the corresponding values of *F* and *M*. Once the system of equations in Equation (1) is solved for *u* and *θ* for each value of the applied voltage (either $V_{R}$ or $V_{L}$), considered to be monotonically increasing, the relevant capacitance change can be obtained. It must be noted that the mentioned system of equations turns out to be nonlinear, as terms *F* and *M* will be next shown to depend nonlinearly on the kinematic variables *u* and *θ*.

### 3.1. Rotational Actuation

In this case, both the stator capacitors are set to an electrical potential $V_{R}$ while the rotor (i.e., the proof mass) is electrically grounded. Under the assumption of the negligible effects of the lateral displacements on the configuration along the capacitor surfaces, the gap $g_{R}$ between the conductors is given by:

(4) $$g_{R}=g_{0}+x_{1}\sin\theta$$

where $g_{0}$ is the initial gap in the absence of any electrostatic actuation, and $x_{1}$ is reported in Figure 4. Since the rotation angle *θ* is supposed to be small (actually less than one degree) for any possible actuation up to pull-in, Equation (4) is assumed to hold true for both the top and bottom capacitors. For this configuration, the shear force is thus zero, i.e.,

(5) $$F_{R}=0$$

while the bending moment reads:

(6) $$\begin{array}{c} M_{R}=-2\int_{a}^{L/2}\frac{\epsilon wV_{R}^{2}}{2{\left(g_{0}+x_{1}\sin\theta \right)}^{2}}\;x_{1}dx_{1}\\ =\frac{\epsilon wV_{R}^{2}}{\sin^{2}\theta }(\frac{\left(g_{0}+a\sin\theta \right)\log\left(g_{0}+a\sin\theta \right)+g_{0}}{g_{0}+a\sin\theta }\\ -\frac{\left(g_{0}+\frac{L}{2}\sin\theta \right)\log\left(g_{0}+\frac{L}{2}\sin\theta \right)+g_{0}}{g_{0}+\frac{L}{2}\sin\theta }) \end{array}$$

where *a* and *L* are the geometric parameters defined in Figure 1 and Figure 4a.

### 3.2. Lateral Actuation

In this case, the rotor is still electrically grounded, while the stator conductor of the lateral capacitor is set to an electrical potential $V_{L}$. Unlike the rotational case, the gap along the lateral surface of the proof mass is not only a function of the rotation angle *θ*, but also of the lateral displacement *u*. Accordingly, the gap can be written as:

(7) $$g_{L}=g_{0}-u-x_{2}\sin\theta$$

where the local coordinate axis $x_{2}$ is shown in Figure 4. The shear force and the bending moment are thus provided as:

(8) $$F_{L}=\int_{0}^{L}\frac{\epsilon wV_{L}^{2}}{2{\left(g_{0}-u-x_{2}\sin\theta \right)}^{2}}\;dx_{2}=\frac{\epsilon wV_{L}^{2}L}{2\left(g_{0}-u\right)\left(g_{0}-u-L\sin\theta \right)}$$

and:

(9) $$\begin{array}{c} M_{L}=\int_{0}^{L}\frac{\epsilon wV_{L}^{2}}{2{\left(g_{0}-u-x_{2}\sin\theta \right)}^{2}}\;x_{2}dx_{2}\\ =\frac{\epsilon wV_{L}^{2}}{2\sin^{2}\theta }\left(\frac{g_{0}-u}{g_{0}-u-L\sin\theta }+\log\left(-g_{0}+u+L\sin\theta \right)-\log\left(-g_{0}+u\right)-1\right) \end{array}$$

### 3.3. Sensing

Since sensing, either the rotational or the lateral one, is independent of the adopted actuation strategy, a discussion is reported next separately.

Neglecting once again the electrical fringe field, the capacitance between the plates is computed through Equation (3), handling the gap *g* as a function of the kinematic variables *u* and *θ* and integrating over the surfaces of the capacitors. In the initial configuration with a gap equal to $g_{0}$ all around the rotor, values of rotational and lateral capacitances respectively read:

(10) $$C_{R0}=2\int_{a}^{L/2}\frac{\epsilon w}{g_{0}}\;dx_{1}=2\frac{\epsilon w(\frac{L}{2}-a)}{g_{0}}\;$$

(11) $$C_{L0}=\int_{0}^{L}\frac{\epsilon w}{g_{0}}\;dx_{2}=\frac{\epsilon wL}{g_{0}}\;$$

In the displaced configuration, they instead amount to:

(12) $$C_{R}=2\int_{a}^{L/2}\frac{\epsilon w}{g_{0}+x_{1}\sin\theta }\;dx_{1}=\frac{2\epsilon w}{\sin\theta }\left(\log\left(g_{0}+\frac{L}{2}\sin\theta \right)-\log\left(g_{0}+a\sin\theta \right)\right)$$

(13) $$C_{L}=\int_{0}^{L}\frac{\epsilon w}{g_{0}-u-x_{2}\sin\theta }\;dx_{2}=\frac{\epsilon w}{\sin\theta }\left(\log\left(-g_{0}+u\right)-\log\left(-g_{0}+u+L\sin\theta \right)\right)$$

## 4. Statistical Effects and Particle Filtering

The analytical model provided in Section 3 is purely deterministic. To infer from the experimental data the possible variability of over-etch and mechanical stiffness of the micro-beam, particle filtering [32,33,34,35,36,37,38] is here adopted.

The effect of variability of the overall Young’s modulus of the polysilicon film on the *C*–*V* plot is ultimately linked to the flexural rigidity of the tested beam. Instead, a variation of the over-etch affects the stiffness of the beam, the actuation terms *F* and *M* and also the measured capacitances by changing the initial gap $g_{0}$. Discriminating the two different sources is not easy, due to the coupled electro-mechanical response of the system: by monitoring the input-output relation at increasing applied voltage and by simultaneously handling both the sensing strategies reported before in an off-line fashion, an estimation of the two stochastic terms is looked for.

Parameter identification of the handled nonlinear system is tackled by using a recursive Bayesian filtering. The sequential importance sampling technique is employed for the simultaneous estimation of the two aforementioned parameters. In this context, the correlation between the sought parameters and the measurement is mathematically expressed via a state-space model. As for the parameters, since they are structure-dependent, they should not vary during a single test at varying loading; anyhow, to allow for model errors induced by the simplified formulae of Section 3 and to update the estimates from presumably wrong initial guesses, a fictitious random walk model is assumed to govern their evolution. If parameters are then collected in a state vector x, its evolution within the interval $\left[t_{k-1}\;\;t_{k}\right]$ representing the (time) window between two subsequent measurements reads:

(14) $$\begin{array}{c} x_{k}=x_{k-1}+v_{k}^{x} \end{array}$$

where $v_{k}^{x}$∼$N(0,Q^{v^{x}})$ denotes a zero mean, white Gaussian noise, whose covariance must be appropriately adjusted to attain accurate estimates. The observation equation is derived from the physical model of Section 3, and it is formally written as:

(15) $$\begin{array}{c} y_{k}=h_{k}\left(x_{k}\right)+w_{k} \end{array}$$

where $y_{k}$ denotes the capacitance measurements at time $t_{k}$; $h_{k}\left(.\right)$ stands for the nonlinear relationship between the parameters in $x_{k}$ and the measurements $y_{k}$; $w_{k}$∼$N(0,Q^{w})$ is the measurement noise.

The inference problem can be regarded as the recursive estimation of the expected value $E[x_{k}|y_{1:k}]$ and/or other statistics of the state vector, conditioned on the observation of the system. Provided that the initial probability distribution function (PDF) of the process $p\left(x_{0}|y_{0}\right)=p\left(x_{0}\right)$ is known, the goal is to estimate $p(x_{k}|y_{1:k})$ upon the availability of the conditional PDF $p(x_{k-1}|y_{1:k-1})$. The problem is often formulated in two distinct stages of prediction and update: in the prediction stage, the Chapman-Kolmogorov integral yields a prior of the state PDF at $t_{k}$ [39]:

(16) $$p\left(x_{k}|y_{1:k-1}\right)=\int p\left(x_{k}|x_{k-1}\right)p\left(x_{k-1}|y_{1:k-1}\right)dx_{k-1}$$

while in the update stage, as soon as the latest observation $y_{k}$ becomes available, Bayes’ rule allows one to correct the PDF through:

(17) $$p\left(x_{k}|y_{1:k}\right)=\varsigma p\left(y_{k}|x_{k}\right)p\left(x_{k}|y_{1:k-1}\right)$$

where *ς* is a normalizing constant, which depends on the likelihood function of the observation process.

To deal with general nonlinear/non-Gaussian problems, sequential Monte Carlo methods [40] can be resorted for evaluating the Chapman-Kolmogorov integral. To this end, the a posteriori PDF is represented via Dirac delta functions at a set of discrete sample points. Without loss of generality, one can then write [41]:

(18) $$p\left(x_{k}|y_{k}\right)=\int p\left(\varepsilon_{k}|y_{k}\right)\delta \left(\varepsilon_{k}-x_{k}\right)d\varepsilon_{k}$$

where δ(.) denotes the Dirac delta function. Assuming that the true posterior $p(x_{k}|y_{k})$ is known and can be sampled, an approximate estimate can be given by:

(19) $$p\left(x_{k}|y_{k}\right)\approx \frac{1}{N_{s}}\sum_{i=1}^{N_{s}}\delta (x_{k}-x_{k}^{i})$$

where $x_{k}^{i}$ is a set of $N_{s}$ random samples drawn from true posterior $p(x_{k}|y_{k})$. However, it is impossible to efficiently sample from the true posterior, as the goal of the procedure is the definition of the true posterior itself. Importance sampling has been introduced as a remedy to this deadlock: an arbitrarily-chosen distribution $\pi (x_{k}|y_{k})$, called the importance function, is sampled in the absence of the true posterior. An unbiased estimate of $p(x_{k}|y_{k})$ can then be obtained as:

(20) $$p\left(x_{k}|y_{k}\right)\approx \sum_{i=1}^{N_{s}}{\tilde{\omega }}_{k}^{i}\delta \left(x_{k}-x_{k}^{i}\right)$$

where ${\tilde{\omega }}_{k}^{i}=\omega_{k}^{i}/\sum_{j=1}^{N_{s}}\omega_{k}^{j}$ and the so-called importance weights $\omega_{k}^{i}$ are approximated through:

(21) $$\omega_{k}^{i}=\frac{p\left(y_{k}|x_{k}^{i}\right)p\left(x_{k}^{i}\right)}{\pi (x_{k}^{i}|y_{k})}$$

and updated through:

(22) $$\omega_{k}^{i}=\omega_{k-1}^{i}\frac{p\left(y_{k}|x_{k}^{i}\right)p\left(x_{k}^{i}|x_{k-1}^{i}\right)}{\pi (x_{k}^{i}|x_{k-1}^{i},y_{1:k})}$$

Algorithmic issues may arise, as the covariance of the weights always increases over time [42]. In practice, after a few iterations, all of the particles but one display negligible weights; this issue is referred to as sample degeneracy [40]. To alleviate sample degeneracy, particle filters are typically complemented with a sequential importance sampling and resampling stage; see, e.g., [43]. During the resampling stage, particles with relatively low probability are deleted, and the ones with higher likelihood are duplicated. Several procedures have been proposed in the literature for resampling, such as: multinomial resampling; stratified resampling; residual resampling; and systematic resampling. For the implementation and algorithmic details, readers are referred to [44]. Using extensive Monte Carlo simulations, it was shown in [45] that, in terms of resampling quality and computational complexity, systematic resampling is favorable; therefore, in this study, the latter resampling scheme is adopted. Resampling may however cause another issue called sample impoverishment, when few particles with a notable weight are kept while all others with a negligible weight are abandoned [46]. In general terms, it can be said that sample degeneracy is a result of samples being too distributed (also in zones with very low probability); instead, sample impoverishment is (as a possible direct negative consequence of resampling) the problem of having the particles be too focused. Sample degeneracy and sample impoverishment are arguably two main drastic flaws associated with particle filters and importance sampling, which together provide unsatisfactory particle distributions [47]. One of the first remedies for mitigating the impoverishment issue was roughening (also called jittering [48], diffusing [49], diversifying [50]), which basically adds an independent Gaussian jittering noise with zero mean and constant covariance to the resampled particles. All of these issues motivated the research on the so-called particle distribution optimization schemes, such as the applications of artificial intelligence methods (e.g., particle swarm optimization, genetic algorithms) and machine learning approaches (e.g., clustering, merging); for a recent review of the state-of-the-art for the subject, see [47]. For the current application, it will be seen in Section 5 that, due to the relatively low number of iterations, sample impoverishment does not prevail, and only a resampling stage has been implemented.

## 5. Results and Discussion

In this section, we first explore the sensitivity of the device response to the two parameters to be identified, namely the over-etch *O* and the Young’s modulus *E* of the film. Next, the particle filter of Section 4 is adopted to estimate the parameter values for each sample. As for the target values $\bar{O}$ and $\bar{E}$ of parameters, we have assumed $\bar{O}=0$, which means that the real geometry of the device matches the design one; $\bar{E}=149.3$ GPa, which is the average between the two (upper and lower) bounding estimates of the homogenized polysilicon Young’s modulus; see [5].

Both *O* and *E* affect the stiffness terms in Equation (1). Specifically, the over-etch comes into play by changing the width of the beam according to $h^{*}=\bar{h}-2O$; further to that, the over-etch also has a role in actuation and sensing, since the gap is affected according to $g_{0}^{*}={\bar{g}}_{0}+2O$. In these equations, the starred values represent the actual geometric features of each sample, while the over-barred ones are the target values (reported in Table 1).

**Figure 5:**
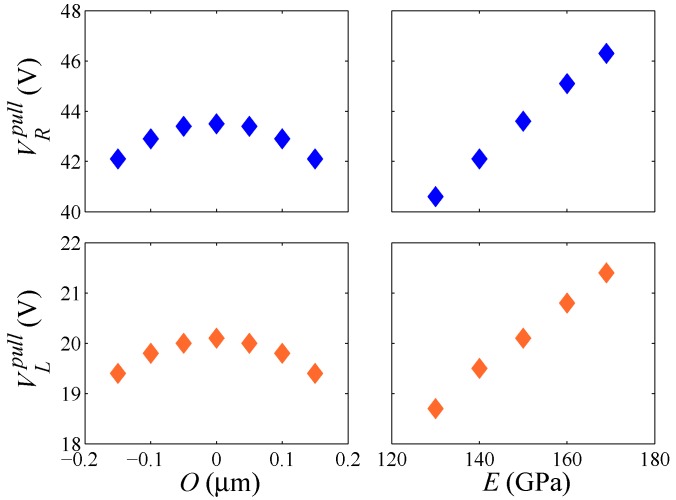
Sensitivity of the pull-in voltages $V_{R}^{pull}$ and $V_{L}^{pull}$ to a variation of (**left**) over-etch *O* or (**right**) the polysilicon Young’s modulus *E*.

**Figure 6:**
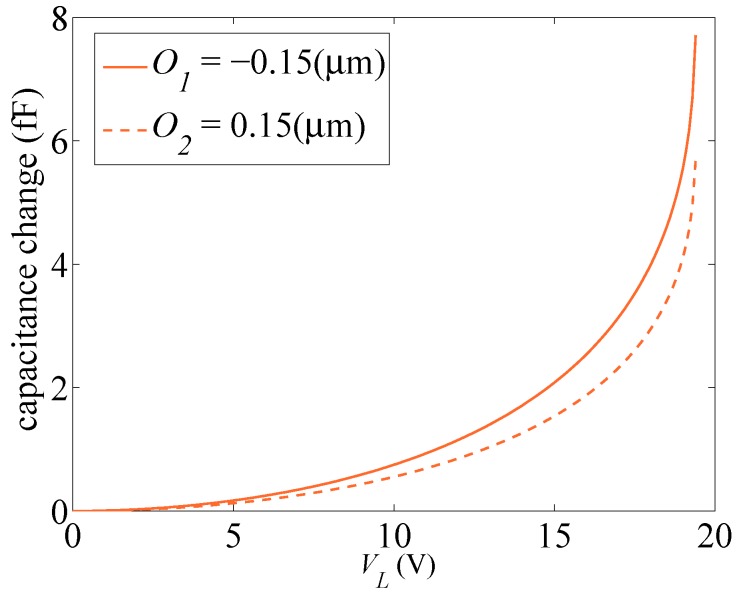
Comparison between the electro-mechanical responses of two devices respectively featuring $O_{1}=-0.15$ μm (solid line) and $O_{2}=0.15$ μm (dashed line), with $E_{1}=E_{2}=\bar{E}$.

For some test structures (see, e.g., [20,21,22]), the pull-in voltage $V^{pull}$ has been suggested as a possible feature of the overall structural response providing insights into the micromechanical details of the device, on top of all of the over-etch. Figure 5 gathers the values of the voltage corresponding to the pull-in instability in the case of either lateral or rotational actuation. The sensitivity to *E* and *O* is numerically assessed by fixing one of the two parameters at the relevant target value (either $\bar{E}$ or $\bar{O}$) and changing the other within the domain E∈ [$E_{min}$ = 130 GPa, $E_{max}$ = 169 GPa] or O∈ [$O_{min}$ = –0.15 μm, $O_{max}$ = 0.15 μm]. It can be noted once again that the lateral actuation is associated with lower pull-in voltages; such an effect has been also observed in the experimentally-measured values, see Figure 2 and Figure 3. A variation of the Young’s modulus is shown to affect the pull-in voltage almost linearly, while the over-etch has a nonlinear impact. This observation is essential if one aims to characterize the device by using the pull-in voltage only, since that would require a one-to-one relation between the varying parameter and $V^{pull}$ [21]. This issue is further illustrated in Figure 6, through the whole response of the system for two different values of *O* leading to very similar values of the pull-in voltage. As can be seen, although the same asymptotic response occurs at pull-in instability, the devices feature a much different capacitance variation in the stable regime. Therefore, the whole response of the system has to be accounted for to correctly characterize the uncertainties.

In the filtering procedure, the Young’s modulus and the over-etch are allowed to vary in the domains considered for Figure 5. The extreme values for the Young’s modulus have been selected according to the range of variation of *E* for single-crystalline silicon (see [51]); the values for the over-etch have been instead assumed on the basis of fabrication process parameters.

Since the adopted Bayesian filtering procedure needs to be initialized with a guess for the handled parameters, coefficients $\xi_{O}$ and $\xi_{E}$ are introduced to allow such initialization values $O_{i}$ and $E_{i}$ to vary around the target ones $\bar{O}$ and $\bar{E}$ according to:

(23) $$O_{i}=O_{min}+\xi_{O}\left(O_{max}-O_{min}\right),\;E_{i}=E_{min}+\xi_{E}\left(E_{max}-E_{min}\right)$$

where $\xi_{O}$ and $\xi_{E}$ then belong to the range [0,1].

**Figure 7:**
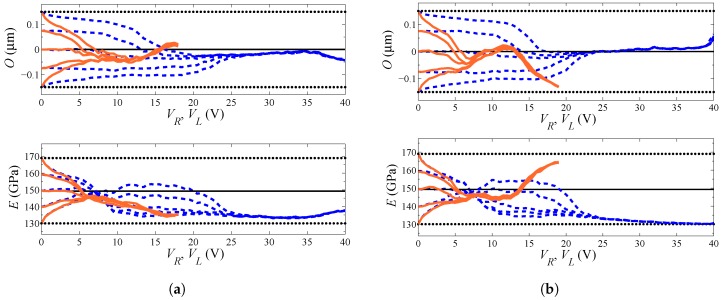
Evolution of the estimates of (**top**) *O* and (**bottom**) *E*, at varying filter initialization. Blue dashed lines: rotational actuation through $V_{R}$; orange solid lines: lateral actuation through $V_{L}$. (**a**) Specimen #2, leading to consistent final estimates, and (**b**) Specimen #5, leading instead to non-consistent results.

Figure 7 shows the evolution of the estimations starting from different initial guess values $O_{i}$ and $E_{i}$, for two different dies. As far as filtering is concerned, in the analyses, 1000 particles have been adopted, and the experimental data have been fed to the filter every 0.5 V. The process noise covariance matrix $Q^{v^{x}}$, linked to the noise term $v_{k}^{x}$ in Equation (14), has been set as $Q^{v^{x}}=\text{diag}\left[\sigma_{v_{O}}^{2}\;\sigma_{v_{E}}^{2}\right]$, so to handle uncorrelated uncertainties related to the geometry and the film morphology and to allow both parameters to evolve within the a priori defined domain of variation. The measurement covariance matrix $Q^{w}$, linked instead to the term $w_{k}$ in Equation (15), has been set as $Q^{w}=\text{diag}\left[\sigma_{w_{R}}^{2}\;\sigma_{w_{L}}^{2}\right]$ to account for the uncertainties associated with experimental measurements. As far as the results here reported are concerned, the following values for the process and measurement noise covariances have been adopted: $\sigma_{v_{O}}^{2}=10^{-4}\;\mu$m${}^{2},\;\sigma_{v_{E}}^{2}=10^{-3}\times {\bar{E}}^{2},\;\sigma_{w_{R}}^{2}=\sigma_{w_{L}}^{2}=10^{-3}$ fF${}^{2}$. Graphs in Figure 7 show results obtained with the lateral actuation (orange lines) superposed onto those obtained with the rotational actuation (blue lines), although relevant to two different series of filter runs. It can be seen that, independently of the test case and sample considered, the final estimates of *O* and *E* are only marginally affected by the initialization values; accordingly, the filter is proven to properly handle the information brought by measurements, without any kind of instability. As the actuation voltage is increased, the ratio between the response of the device (in terms of capacitance change) and the measurement errors is also increased (see Figure 2 and Figure 3): a more accurate parameter estimation is thus expected. This is somehow reported in Figure 7, since the estimates, starting from different initialization points, first follow different paths, and then, all merge to provide the mentioned initialization-independent solutions. Since the effects of actuation are bigger in the lateral case, the merging of all of the curves is shown to happen in this case for smaller values of the applied voltage.

Parameter identification has been carried out for all ten samples mentioned in Section 2, whose *C*–*V* plots are reported in Figure 2 and Figure 3, and the relevant final values of the estimates of *O* and *E* are reported in Table 2. Results can be classified into two sets: those featuring final estimations for the two kinds of actuation matching well each other in the proposed statistical setting; and those providing rather different estimations with the two types of actuation, in terms of either of the two parameters. Graphs in Figure 7a, referring to Specimen #2, are exemplary of an actuation-independent solution, although the estimations of *O* are somehow diverging close to pull-in. Graphs in Figure 7b, referring to Specimen #5, show instead the pathological divergence of the two solutions, in terms of both *O* and *E*, once again maximized close to pull-in.

**Figure 8:**
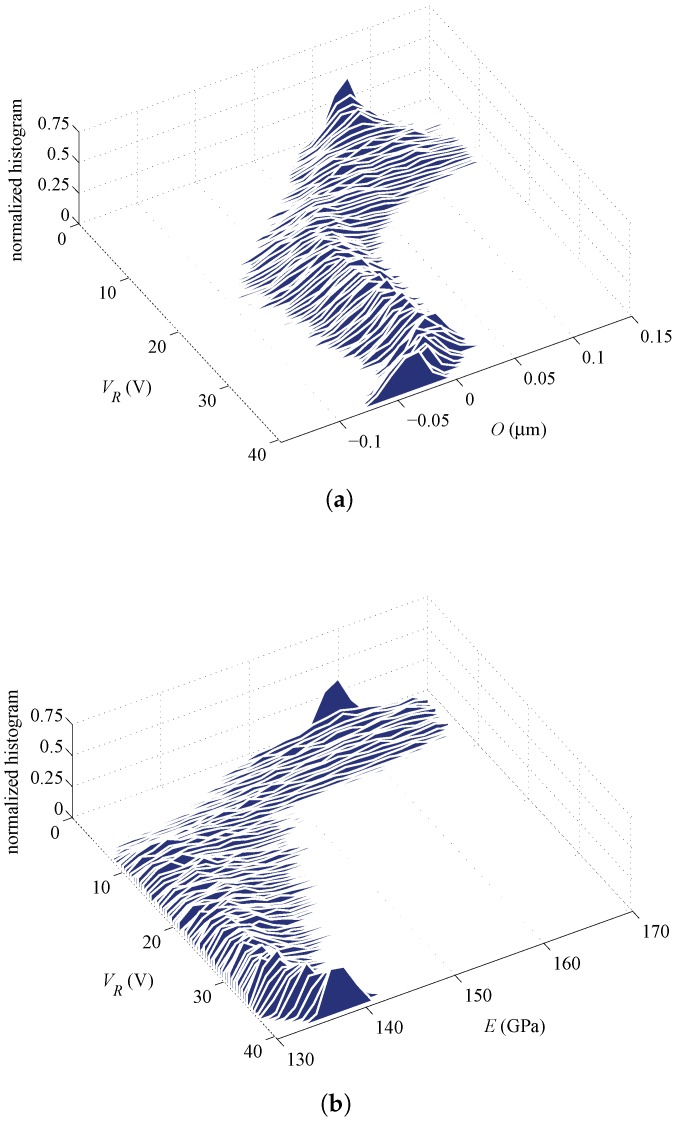
Specimen #2, rotational actuation case, $\xi_{O}=\xi_{E}=0.75$: evolution of the PDFs of (**a**) *O* and (**b**) *E* corresponding to the evolution of estimates represented by blue curves in Figure 7a.

**Figure 9:**
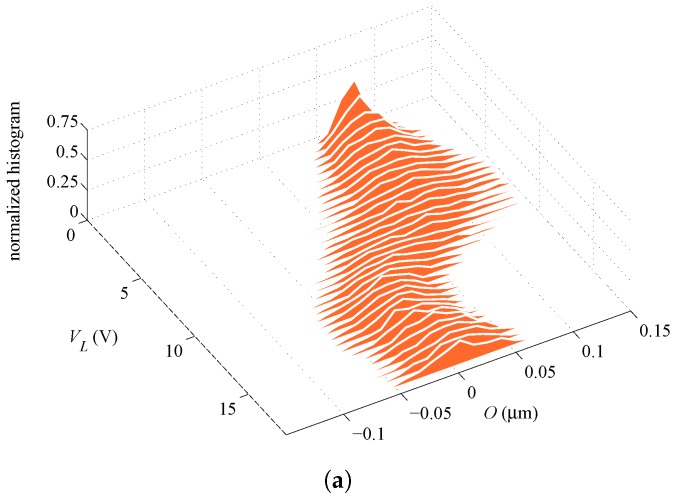

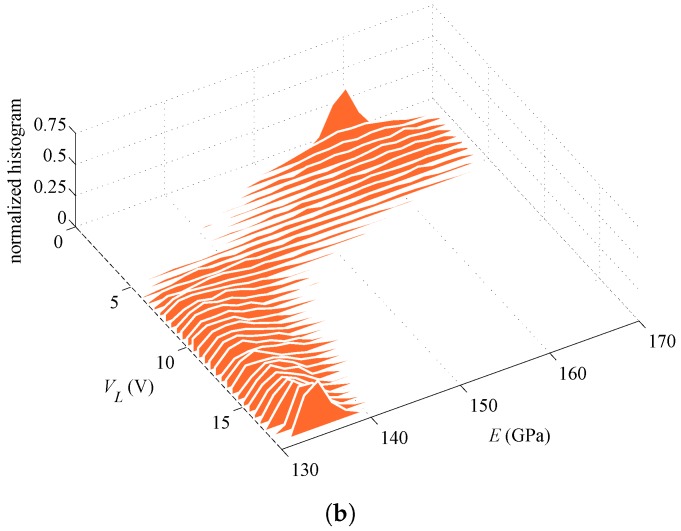
Specimen #2, lateral actuation case, $\xi_{O}=\xi_{E}=0.75$: evolution of the PDFs of (**a**) *O* and (**b**) *E* corresponding to the evolution of estimates represented by orange curves in Figure 7a.

**Figure 10:**
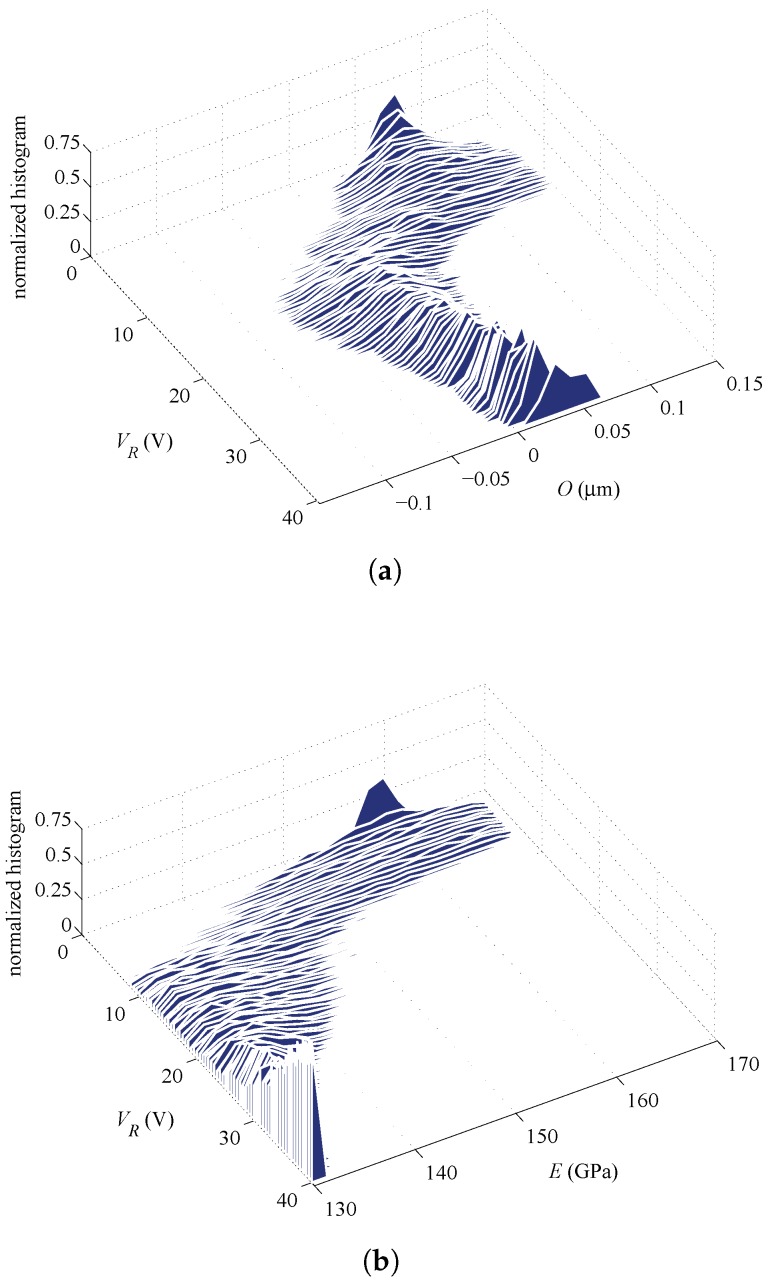
Specimen #5, rotational actuation case, $\xi_{O}=\xi_{E}=0.75$: evolution of the PDFs of (**a**) *O* and (**b**) *E* corresponding to the evolution of estimates represented by blue curves in Figure 7b.

**Figure 11:**
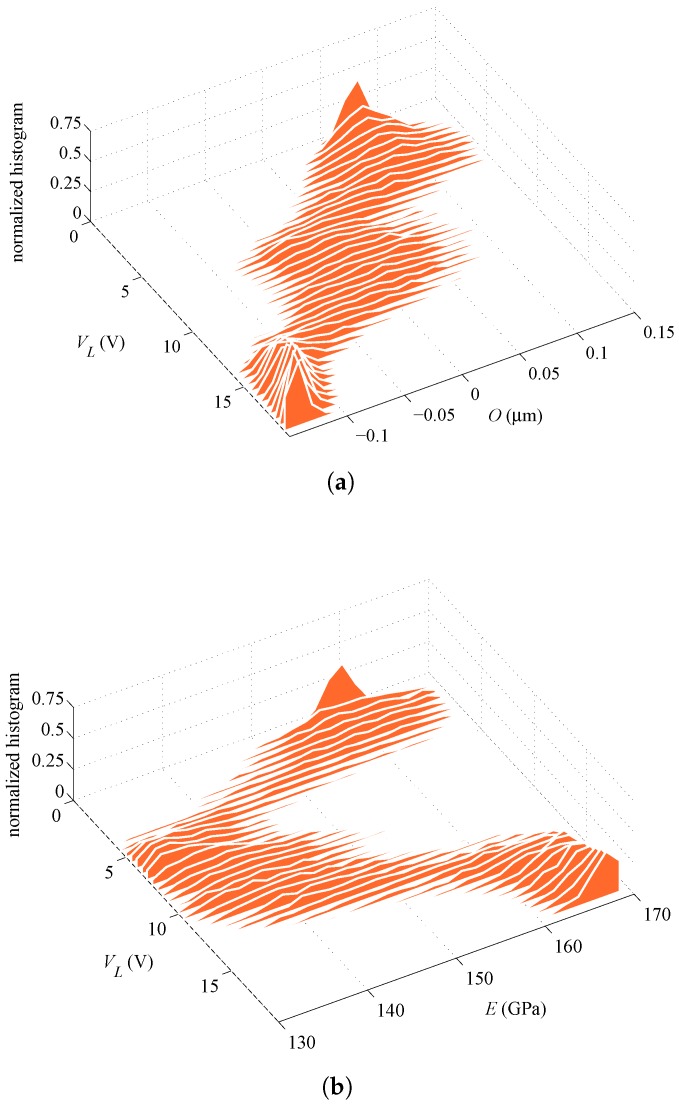
Specimen #5, lateral actuation case, $\xi_{O}=\xi_{E}=0.75$: evolution of the PDFs of (**a**) *O* and (**b**) *E* corresponding to the evolution of estimates represented by orange curves in Figure 7b.

In addition to the average or expected values reported above, further insights are provided next in terms of the evolution of the PDFs of the parameters during the whole filtering procedure. Figure 8 and Figure 9 gather the evolution of PDFs of the parameters for Specimen #2, with an initialization at $\xi_{O}=\xi_{E}=0.75$, for the rotational and lateral actuation, respectively; these results correspond to the evolution of averages shown in Figure 7a. As can be seen, the parameters show a similar evolution of the PDFs as the actuation voltage increases up to pull-in, independently of the actuation type. The relatively large superposition of the PDFs of the estimated parameters at the final stages of the procedure, as provided by the rotational and lateral actuations, is basically pointing towards a common statistical solution for the parameters. On the other hand, the PDFs of *O* and *E* in Figure 10 and Figure 11, which correspond to Specimen #5 and to the same initialization at $\xi_{O}=\xi_{E}=0.75$, show no superposition with the two types of actuation. Hence, though the procedure is algorithmically stable for each actuation type, estimates do not conform in a statistical sense and, so, do not offer a unique solution.

Although the filtering procedure has been shown to be robust, some samples led to different estimates of *E* and/or *O* depending on the actuation or estimates located at the bounds of the initial variation range. Hence, the adopted model might not be accurate enough for all of the possible situations encountered: while an effective homogeneous Young’s modulus for the polysilicon beam can be appropriate to describe the overall stiffness of each beam (see [4,5,29]), the assumption of a uniform over-etch all around the plate and the beam may fail for different dies in a single wafer. Furthermore, small imperfections, like, e.g., non-zero values of *u* and *θ* at no actuation (i.e., for $V_{L}=V_{R}=0$), could lead to a drift or bias in the solution obtained with the particle filter. It has been also assumed that the beam anchors behave ideally, even if several studies have shown that stress absorption and deformation at the anchors [52,53] can induce an additional compliance and cause discrepancies between the model and measured responses. The effects of these additional sources of uncertainty at the micron or even sub-micron scale will be considered in future investigations.

## 6. Conclusions

In this paper, we have provided a stochastic analysis of the effects of micromechanical uncertainties on the response of a polysilicon film. Through on-chip tests on specimens featuring a constant slenderness and a small ratio (on the order 3–4) between their width and the characteristic size of silicon grains, the scattering in the overall response in terms of capacitance change vs. applied voltage (*C*–*V*) has been assessed. An analytical reduced-order model has been formulated based on standard (Cauchy) continuum elasticity, beam kinematics and electrostatics in narrow domains between parallel or slightly tilted plates.

Geometrical imperfection and polycrystalline morphology effects have been accounted for through fluctuations of the over-etch *O* and of the overall Young’s modulus *E* of the film, respectively. Their sample-dependent expected values have been estimated via a particle filtering approach, designed to exploit the whole *C*–*V* response measured experimentally (almost) up to pull-in. To collect measurements with a certain degree of redundancy, two different types of actuation and sensing have been designed, inducing two loading conditions on each sample. Results of the identification procedure have shown that the reported scattering in the *C*–*V* curves can be linked to somehow different values of *O* and *E* for specimens taken from different dies in a wafer. A thorough analysis has been presented via the proposed approximate analytical model, formulated to interpret the experimental data. Such a simplified model missed the effects of: the non-uniform distribution of the over-etch; initial imperfections; anchor compliance. The mentioned effects will be considered in future investigations, to better assess the possible impact of micromechanical features on the response of micromechanical systems.

## Figures and Tables

**Table 1:** Geometric dimensions of the device.

Parameter	Value	Parameter	Value
beam length (*l*)	20 μm	initial gap at capacitors ($g_{0}$)	2 μm
beam width (*h*)	2 μm	*a*	17 μm
out-of-plane film thickness (*w*)	22 μm	plate sidelength (*L*)	200 μm

**Table 2:** Estimated parameter values through particle filtering.

	*O* (μm)			*E* (GPa)	
Specimen #	Through $V_{R}$	Through $V_{L}$		Through $V_{R}$	Through $V_{L}$
1	−0.13	−0.13		138.4	131.8
2	−0.04	0.02		137.4	135.3
3	0.03	−0.09		134.3	151.9
4	−0.12	−0.07		145.7	132.1
5	0.06	−0.13		130.5	164.2
6	0.01	0.00		144.5	166.3
7	−0.14	−0.15		136.5	130.4
8	−0.14	−0.15		140.1	130.1
9	−0.15	−0.15		159.1	130.1
10	−0.15	−0.15		152.8	130.2
